# Low diagnostic yield and time to diagnostic confirmation results in prolonged use of antimicrobials in critically ill children

**DOI:** 10.12688/wellcomeopenres.16848.2

**Published:** 2022-03-03

**Authors:** John Clark, Deborah White, Esther Daubney, Martin Curran, Rachel Bousfield, Theodore Gouliouris, Elizabeth Powell, Adam Palmer, Shruti Agrawal, David Inwald, Iain Kean, M. Estée Török, Stephen Baker, Nazima Pathan

**Affiliations:** 1 Department of Paediatrics, University of Cambridge, Cambridge, Cambridgeshire, CB2 0QQ, UK; 2 Cambridge University Hospitals NHS Foundation Trust, Cambridge, Cambridgeshire, CB2 0QQ, UK; 3 Clinical Microbiology and Public Health Laboratory, Public Health England, Cambridge, Cambridgeshire, CB2 0QQ, UK; 4 Department of Medicine, University of Cambridge, Cambridge, Cambridgeshire, CB2 0QQ, UK; 5 Cambridge Institute of Therapeutic Immunology and Infectious Disease, University of Cambridge, Cambridge, Cambridgeshire, CB2 0QQ, UK

**Keywords:** Paediatric intensive care units, infections, anti-infective agents, microbial drug-resistance, microbiological techniques, routine diagnostic tests

## Abstract

**Background:** Broad-spectrum antimicrobial therapy is a key driver of antimicrobial resistance. Here, we aimed to review indications for antimicrobial therapy, determine the proportion of suspected bacterial infections that are confirmed by culture, and assess the time taken for microbiology test results to become available in the paediatric intensive care unit (PICU).

**Methods**: A single-centre prospective observational cohort study of 100 consecutive general PICU admissions from 30 October 2019 to 19 February 2020. Data were collected from the hospital medical record and entered into a study database prior to statistical analysis using standard methods.

**Results**: Of all episodes of suspected infection, 22% of lower respiratory tract infection, 43% of bloodstream and 0% of central nervous system infection were associated with growth on microbiology culture. 90% of children received antimicrobial therapy. Hospital-acquired infection occurred less commonly than primary infection, but an organism was grown in a greater proportion (64%) of cultures. Final laboratory reports for negative cultures were issued at a median of 120.3 hours for blood cultures and 55.5 hours for endotracheal tube aspirate cultures.

**Conclusions**: Despite most critically children receiving antimicrobial therapy, infection was often not microbiologically confirmed. Novel molecular diagnostics may improve rationalisation of treatment in this population.

## Introduction

The annual number of infections caused by antimicrobial resistant (AMR) organisms in England is >70,000 ^1^. Optimised prescribing practice has been highlighted as a key national strategy to combat AMR ^2^. However, it is challenging for clinicians to reduce the use of broad spectrum antimicrobials when faced with severe and undifferentiated illness encountered in the paediatric intensive care unit (PICU). The timely administration of antimicrobials is paramount, as survival decreases 7.6% for every hour antimicrobial therapy is delayed in patients with septic shock ^3^. Clinical prediction scores for infection perform poorly in children ^4, 5^, and there is a reliance on diagnostic tests to assist in rationalisation of antimicrobial therapy. Here we aimed to better understand the impact of microbiology results and antimicrobial prescribing in a PICU in a major UK tertiary referral centre as previous reports have been limited to point prevalence studies and hospital-acquired infection (HAI) alone.

## Methods

This study included 100 patients admitted to the PICU at Cambridge University Hospitals NHS Foundation Trust between 30 October 2019 and 19 February 2020. The sample size was based on the estimated number of ventilated PICU admissions based on previous PICANet data over a four month study period ^6^. This allowed completion of this project prior to commencement of an interventional study relating to rapid diagnostic testing (Rapid Assay for Sick Children with Acute Lung infection Study, ClinicalTrials.gov: NCT04233268, 18/01/2020). Formal sample size calculation was not indicated given the descriptive and exploratory nature of the study. All admitted patients were reviewed for eligibility during the enrolment period. Patients were identified by daily screening of the electronic medical record (Epic, Verona, USA) by the lead, medically qualified author by reviewing the active PICU admission patient list.

 Data were entered into a study database formatted for this project on an electronic data capture system (REDCap 9.5.19) hosted by University of Cambridge ^7, 8^. This database allowed data to be checked at the time of entry through customisation of accepted data into fields, for example, placing permissible date-time ranges for entry only during the intended study period. The project was registered on the Addenbrooke’s Hospital Quality and Safety Information System (QSIS, project ID 2606, 13 November 2019). This registration facilitated internal review within the hospital, and the project was authorised as a clinical audit. As no identifiable patient data or intervention was a component of the study, this authorised the project to take place and ethics review was not required. Given data was de-identified, waiver of consent was approved in this review.

Patients were eligible for inclusion in the study if they received mechanical ventilation via an endotracheal tube (ETT) during their admission and were aged ≥37 weeks corrected gestation at the time of enrolment. These criteria were used to avoid inclusion of patients who were not critically unwell and receiving high dependency level care only, and patients that would have otherwise gone to neonatal intensive care except for capacity and cohorting related reasons. Antimicrobial decision making in this institution is directed by internal prescribing guidelines produced by the antimicrobial stewardship team (available on request from lead author), with deviation from these guidelines on consultation with the microbiology team and review of investigations and the clinical situation. Broadly, first line treatment for severe community acquired pneumonia is ceftriaxone (for children aged greater than one month) with consideration of atypical cover with azithromycin or clarithromycin. Co-amoxiclav is used for aspiration pneumonia, and piperacillin-tazobactam for suspected ventilator associated pneumonia. Community acquired septicaemia is treated with ceftriaxone and gentamicin (in children greater than one month) whilst piperacillin-tazobactam and gentamicin is used for children with hospital-acquired septicaemia. Ceftriaxone or cefotaxime is the first line treatment for community acquired meningitis, with the addition of aciclovir for encephalitis.

Clinical diagnostic samples were submitted to the on-site Public Health England Clinical Microbiology and Public Health Laboratory and processed according to the UK Standards for Microbiology Investigations ^9^. The PICU is supported by a clinical microbiology team who provide a 24 hour on call service in addition to microbiology rounds that take place twice a week.

Where a patient had investigations undertaken at an external hospital prior to PICU admission, data were collected from the inter-hospital transfer notes, and documented communications between PICU and the referring hospital. All documented investigations to a maximum period of one week prior to PICU admission were included in this study. Turnaround times from referring centres were not included in the study, due to the various structures and staffing of other hospitals. In addition, this would require a formal consent process to access the external medical record.

The primary indication for admission was determined through chart review. It was defined as the main organ system or disease process for which intensive care admission was required. A patient was considered to have ventilator-associated pneumonia (VAP) if antimicrobial therapy for lower respiratory tract infection (LRTI) was commenced ≥48 hours following PICU admission. This definition was selected because of the known poor specificity of paediatric diagnostic scores for VAP ^4^. Sepsis was defined as per the Goldstein criteria ^10^. A suspected infection was defined by the commencement of antimicrobial therapy by the clinical team. Each type of suspected infection was evaluated individually due to the issues of secondary infection, multi-compartment infection and new infections occurring through the admission to PICU.

Statistical analysis was performed using SPSS (version 24) ^11^. Simple descriptive statistics were used to describe counts and percentages. Given non-normality of the datasets, median and interquartile ranges were reported.

## Results

### Baseline patient characteristics

Between 18 November 2019 and 19 February 2020 there were 210 admissions to the PICU. Of these, three were excluded because their age was <37 weeks corrected gestational age and 107 were excluded because they did not require mechanical ventilation. Of the 100 admissions that fulfilled the inclusion criteria, 78 were single admissions and 22 were re-admissions ^12^. There were 55 (71%) male patients, comprising 61 (61%) of admissions. The median age was 11.2 months (interquartile range (IQR), 2.2 to 58.0 months). The median weight was 8.0kg (IQR, 4.1 to 20.4kg). There were 94 emergency admissions and six elective admissions. The majority of patients were direct transfers to PICU from external hospitals (n = 66) with the remainder being from the Emergency Department (n = 14), transfers from the wards (n = 11) and Theatre and Recovery Area (n = 9). The median Paediatric Index of Mortality 3 (PIM3) score ^13^ was 0.97 (IQR, 0.46 to 3.49). The median length of stay in the PICU was five days (IQR, two to eight days), with a total 747 PICU admission days; 94% of children survived and were discharged.

### Patients with suspected and confirmed infection

Respiratory problems were responsible for 49% of admissions, of which 63% had bronchiolitis, 18% pneumonia, 10% structural airway problems, 4% asthma, 2% congenital diaphragmatic hernia, and 2% mediastinal mass ( Table 1). The most common indications for commencing antimicrobials within 48 hours of admission were suspected LRTI (52%), central nervous system (CNS) (29%) and bloodstream infection (19%) ( Table 2). The number of episodes of treatment exceeds the number of admissions due to some patients having multi-organ infection such as sepsis secondary to LRTI. For some patients, whilst an infection was treated it did not represent the primary indication for admission to PICU. Microbiological culture confirmed the presence of a bacterial or fungal organism in 17% of cases of suspected LRTI and 37% of cases of suspected bloodstream infection. No microbiological diagnosis was made for other suspected primary infections.

**Table 1.** Indications for admission to paediatric intensive care.

Primary indication for admission	Number of admissions
Respiratory	49
Bronchiolitis	31
Pneumonia	9
Structural airway problem	5
Asthma	2
Congenital diaphragmatic hernia	1
Mediastinal mass	1
Neurological	36
Sepsis	2
Routine post-operative	5
Cardiac	4
Trauma	3
Acute lymphocytic leukaemia	1
Total	100

**Table 2.** Proportion of suspected infections treated with antimicrobial therapy confirmed by growth on culture.

Type of suspected infection treated with antimicrobial therapy	Culture site	Growth on culture/Total episodes of treatment (%)		
		<48 hours PICU admission	≥48 hours PICU admission	**Total**
Blood stream	Blood culture	7/19 (37)	2/2 (100)	**9/21 (43)**
Central nervous system	Cerebrospinal fluid	0/29 (0)	0/1 (0)	**0/30 (0)**
Intra-abdominal	Aspirate of collection	0/3 (0)	0/0 (0)	**0/3 (0)**
Line	Line tip	0/0 (0)	3/3 (100)	**3/3 (100)**
Lower respiratory	Tracheal aspirate	9/52 (17)	4/7 (57)	**13/59 (22)**
Soft tissue	Wound swab	0/2 (0)	0/1 (0)	**0/3 (0)**
**Total**		**16/105 (15)**	**9/14 (64)**	

PICU, paediatric intensive care unit.

PICU-acquired infection occurred less commonly (n = 14), but a bacterial isolate was identified in a greater proportion of HAIs than community-acquired infections. ( Table 2). There were seven instances of hospital-acquired pneumonia (six ventilator-associated pneumonia) of which four were associated with a bacterial isolate. This equates to a suspected ventilator associated pneumonia rate of 10.3 per 1000 ventilator days (6 cases for 580 days of mechanical ventilation). There were two episodes of treatment for presumed bloodstream infection, and three episodes of presumed line infection, all with positive microbiological cultures. This equates to a central line infection rate of 7.8 per 1000 central line days (3 cases from 383 central line days).

There were no positive cultures in patients with suspected CNS infection (n = 1) or skin and soft tissue infection (n = 1).

### Performance of microbiology and virology investigations prior to PICU admission

 Of the 100 PICU admissions, 50 had microbiology and virology tests obtained prior to PICU admission. This encompassed 81 investigations for infection. The most common investigations were blood culture (35/81 tests) and nasopharyngeal aspirate for respiratory viruses (25/81 tests) ( Table 3).

**Table 3.** Microbiology and virology tests obtained prior to PICU admission.

Test	Number of samples	Tested positive n (%)
Blood culture	35	5 (14)
CSF culture	5	0
CSF viral PCR	1	0
ETT aspirate culture	1	1 (100)
Fluid culture (intra-abdominal)	1	0
NPA viral PCR	25	23 (92)
NP swab viral PCR	9	6 (67)
Sputum culture	3	2 (67)
Wound swab culture	1	1
**Total**	81	38 (47)

NP Nasopharyngeal; NPA: nasopharyngeal aspirate; CSF: cerebrospinal fluid; PCR: polymerase chain reaction; ETT: endotracheal tube; PICU: paediatric intensive care unit.

### Performance of microbiology and virology investigations undertaken during PICU admission

Of the 93 admissions in which cultures were performed, 81 (87%) had samples collected in the PICU following antimicrobial therapy, five (5%) had samples taken before antimicrobial therapy, and seven (8%) had a culture taken before and after antimicrobial therapy. The most common samples were ETT aspirates, blood and cerebrospinal fluid ( Table 4). The median times for the laboratory to report the organism identity and antimicrobial susceptibilities for ETT aspirates and blood cultures were 55.0 hours and 102.5 hours, respectively. There were insufficient samples in the study to provide summary data for time to organism identity and susceptibility for urine specimens. The median times for a final report of no growth on cultures from time of receipt of specimens were 55.5 and 120.3 hours for ETT aspirates and blood cultures, respectively.

**Table 4.** Microbiology tests and turn-around times.

Test	Number of samples	Tested positive n(%)	Time in hours from laboratory receipt of specimen to organism identity and bacterial susceptibilities (median, IQR)	Time in hours from laboratory receipt of specimen to final report of a negative result (median, IQR)
Blood culture	79	8 (10)	55.0 (43.5 – 72.0)	120.3 (120.2 – 121.0)
CSF culture	41	0 (0)	N/A	44.2 (42.0 – 49.9)
CSF viral PCR	25	0 (0)	N/A	39.3 (26.4 – 41.2)
ETT aspirate culture	85	30 (35)	102.5 (74.8 – 153.5)	55.5 (50.9 – 71.1)
ETT viral PCR	20	10 (50)	21.7 (19.7 – 23.4)	55.0 (38.2 – 64.6)
NPA viral PCR	79	62 (78)	21.6 (15.2 – 25.7)	23.6 (21.1 – 24.5)
NP swab viral PCR	20	16 (80)	24.4 (20.2 – 29.3)	23.3 (17.2 – 26.5)
Urine culture	39	4 (10)	*	5.7 (2.7 – 13.8)
**Total**	388	130 (34)		

ETT: endotracheal tube; IQR: interquartile range; N/A: not applicable; NP: nasopharyngeal; NPA: nasopharyngeal aspirate; CSF: cerebrospinal fluid; PCR, polymerase chain reaction. * Insufficient sample size.

Respiratory virus PCR results were returned after a median 21.7 hours on ETT aspirates, 21.6 hours on nasopharyngeal aspirates (NPA) and 24.4 hours on nasopharyngeal (NP) swabs. Of the respiratory samples tested for viruses, a pathogen was detected in 74% of tests, of which respiratory syncytial virus (RSV) was the most common ( Table 5). The most commonly detected bacterial species on ETT aspirate culture and Non-Bronchoscopic Bronchoalveolar Lavage (NB-BAL) were *Staphylococcus aureus* (11% of admissions), *Pseudomonas aeruginosa* (5%), *Enterobacter cloacae* (3%), *coagulase-negative staphylococci* (2%) and *Moraxella catarrhalis* (2%). Other species identified included *Acinetobacter pittii*, *Citrobacter freundii*, *Enterococcus* species, *Escherichia coli*, *Haemophilus influenzae*, *Bordetella pertussis*, *Pseudomonas chloroaphis*, *Staphylococcus haemolyticus*, *Staphylococcus hominis* and *Streptococcus pyogenes*.

**Table 5.** Viral pathogens detected on respiratory samples.

Virus	Endotracheal tube viral PCR N = 20	Nasopharyngeal aspirate viral PCR N = 79	Nasopharyngeal swab viral PCR N = 20	Total N = 119
Respiratory syncytial virus	8	33	7	48
Rhinovirus	2	17	6	25
Enterovirus	0	8	3	11
Adenovirus	1	6	2	9
Human metapneumovirus	0	5	4	9
Parainfluenza	0	4	0	4
Picornaviruses (undifferentiated)	1	2	1	4
Influenza A	1	2	0	3
**Total pathogen detections**	**13**	**77**	**23**	**113**
**Any pathogen detected on test**	**10 (50%)**	**62 (78%)**	**16 (80%)**	**88 (74%)**

PCR: polymerase chain reaction.

At least one antimicrobial was administered prior to 72/100 PICU admissions and during 90/100 PICU admissions. An antimicrobial was given within the first 24 hours of admission in 83% of cases, and within 24 hours of discharge for 50% of admissions. To determine total antimicrobial use, proportion of days on antimicrobial therapy were calculated per days of admission to PICU. Antimicrobials were given for a median 66.7% of days of PICU admission (IQR, 34.6 to 100%). The most commonly prescribed antimicrobials on PICU included beta-lactams (57.7%), macrolides (19.9%) and aminoglycosides (10.7%) ( Table 6).

**Table 6.** Days of antimicrobial therapy by class given to critically ill children.

Antimicrobial class	Total days of treatment	Proportion of total days of PICU admission (%)	Antimicrobial	Total days of treatment *	Proportion of total days of PICU admission (%)
Aminoglycoside	80	10.7	Gentamicin	38	5.1
			Tobramycin	43	5.8
Beta-lactam	431	57.7	Amoxicillin	4	0.5
			Benzylpenicillin	14	1.9
			Cefotaxime	19	2.5
			Ceftazidime	25	3.3
			Ceftolozane - tazobactam	17	2.3
			Ceftriaxone	210	28.1
			Co-amoxiclav	22	2.9
			Flucloxacillin	35	4.7
			Meropenem	53	7.1
			Piperacillin-tazobactam	88	11.8
Fluoroquinolone	31	4.1	Ciprofloxacin	31	4.1
Glycopeptide	37	5.0	Vancomycin	37	5.0
Macrolide	149	19.9	Azithromycin	43	5.8
			Clarithromycin	114	15.3
Other	64	8.6	Chloramphenicol	7	0.9
			Clindamycin	11	1.5
			Co-trimoxazole	12	1.6
			Colomycin	8	1.1
			Doxycycline	1	0.1
			Linezolid	1	0.1
			Metronidazole	10	1.3
			Mupirocin	14	1.9
**Total**	**792**			**857**	

PICU: paediatric intensive care unit. *Note – 20 children were prescribed more than one antimicrobial of the same class on the same day leading to a discrepancy between total number of days of treatment by individual antimicrobial and antimicrobial class.

## Discussion

This study provides insight into antimicrobial prescribing practice and microbiology investigations in a general PICU in a tertiary referral centre in the East of England. The report is unique as previous studies describing microbiology tests and antimicrobial prescribing in critically ill children do not provide a detailed overview of practice for the entire PICU admission. It demonstrates that the majority of ventilated children admitted to PICU receive antimicrobial therapy in the absence of a positive microbiological culture. Of the combined primary and PICU-acquired infections, a bacterial isolate was identified in just 21%. This is consistent with European point prevalence data of paediatric prescribing, which suggest 25.7% of hospital antimicrobial prescriptions are tailored towards known pathogen identity ^14^. In a UK PICU surveillance study, proven infection was found in <7% of children treated with antimicrobials ^15^. Given existing clinical prediction scores are unreliable in PICU, it is important for diagnostic tests to be evaluated.

Microbiological culture techniques are labour-intensive, time consuming, and can take several days to yield a result. In this study, a final negative report was issued at a median of 55.5 hours for ETT aspirate and at 120.3 hours for blood cultures. PICU prescribers will often wait 24–48 hours for any preliminary microbiology findings prior to changing or ceasing antimicrobial treatment. This is reflected in the fact that antimicrobial therapy was administered in 66.7% of all days of PICU admission. For example, the majority of patients admitted to PICU for primary neurological reasons were intubated for status epilepticus; however, it is difficult for clinicians to identify which of these patients have meningoencephalitis, which although uncommon may be catastrophic if untreated. Faster tests to rule out severe infection could reduce the total doses patients require of antimicrobial therapy.

Antimicrobial use was influenced by the high proportion (52%) of admissions treated for severe acute LRTI. Of these admissions, there was growth on ETT aspirate culture in just 17%. This low yield may be due to a number of factors including the sampling technique ^16^, prior antimicrobial therapy and limitations of microbiology culture ^17^. The routine sampling technique for LRTI culture was ETT aspirate. The unit has now shifted towards NB-BAL sampling as this has been demonstrated to have a higher yield and to be a well-tolerated sampling technique ^16, 18^. Cultures may also be negative due to no bacteria being present, hence the value of fast turnaround highly sensitive tests for early cessation of antimicrobial therapy.

VAP occurred in six (6%) of the patients equating to 10.3 cases per 1000 ventilator days, a similar prevalence to another UK study ^19^. This was the most common PICU-acquired infection, which was also the case in a previous three-year retrospective study on this unit ^20^. Of admissions with VAP, four had positive cultures. This is a higher proportion than for primary LRTI, but a larger sample size is needed to demonstrate statistical difference between groups. An organism was identified in 57% of episodes of treatment for PICU-acquired LRTI, similar to the 55.6% detection rate in a previous study ^16^.

Our study is limited by being undertaken in a single centre. The data are likely to under-report presence of bacterial and fungal pathogens and AMR given the limitations of routine culture in detecting pathogens. Microbiology culture performance was likely limited due to the majority of samples in PICU being obtained after antimicrobial therapy, albeit culture yield was also low in pre-PICU samples which are typically obtained prior to antimicrobial therapy in clinical practice. Therefore, the threshold for treatment and factors contributing to commencement of treatment are important to understand. It would be ideal for future studies to occur in multiple centres and over a longer study duration to obtain a better profile of pathogens identified in PICUs in this region.

## Conclusions

Antimicrobials are frequently prescribed in our centre’s PICU for presumed bacterial infection. Bacterial culture has a long turnaround time and may fail to identify potential bacterial pathogens, particularly where antimicrobial therapy is commenced prior to culture. In contrast, in viral PCR testing, a virus was identified in 74% of respiratory samples obtained. Rapid novel bacterial molecular diagnostic techniques could assist clinicians in making a microbiological diagnosis and rationalising broad spectrum antimicrobial therapy earlier. Early, tailored antimicrobial therapy is key to good antimicrobial therapy and the global fight against AMR.

## Data availability

### Underlying data

Open Science Framework: Paediatric intensive care prescribing and infection investigations cohort study. https://doi.org/10.17605/OSF.IO/C6WU5 ^12^.

This project contains the following underlying data:

- Raw data file.CSV
- Data dictionary.CSV

Data are available under the terms of the Creative Commons Zero "No rights reserved" data waiver (CC0 1.0 Public domain dedication).
