# Supplementary material for: Low diagnostic yield and time to diagnostic confirmation results in prolonged use of antimicrobials in critically ill children
Source: Wellcome Open Res. 2022 Mar 3;6:119. Originally published 2021 May 18. [Version 2] doi: 10.12688/wellcomeopenres.16848.2 (PMC8902261; doi:10.12688/wellcomeopenres.16848.2)
Supplement: Peer review history

# Peer review: Reviewer response for version 2

**DOI:** 10.21956/wellcomeopenres.19579.r48995

**Published:** 2022-03-07

**Authors:** Sanjay Patel

**Affiliations:** 1 The British Society for Antimicrobial Chemotherapy, Birmingham, UK

Many thanks to the authors for responding to the comments that I made. I agree that a study looking into the impact of a multiplex bacterial and viral panel on PICU may be helpful in improving AMS in this setting. I look forward to seeing the results of their study.

Is the work clearly and accurately presented and does it cite the current literature?

Partly

If applicable, is the statistical analysis and its interpretation appropriate?

Not applicable

Are all the source data underlying the results available to ensure full reproducibility?

Yes

Is the study design appropriate and is the work technically sound?

Yes

Are the conclusions drawn adequately supported by the results?

No

Are sufficient details of methods and analysis provided to allow replication by others?

Yes

Reviewer Expertise:

Antimicrobial stewardship and AMR

I confirm that I have read this submission and believe that I have an appropriate level of expertise to confirm that it is of an acceptable scientific standard.

# Peer review: Reviewer response for version 2

**DOI:** 10.21956/wellcomeopenres.19579.r48994

**Published:** 2022-03-04

**Authors:** Arne Simon

**Affiliations:** 1 Pediatric Hematology and Oncology, Children's Hospital Medical Center, University Hospital of Saarland, Homburg, Germany

Many thaks for the revised version, no further comments.

Is the work clearly and accurately presented and does it cite the current literature?

Yes

If applicable, is the statistical analysis and its interpretation appropriate?

Yes

Are all the source data underlying the results available to ensure full reproducibility?

No source data required

Is the study design appropriate and is the work technically sound?

Yes

Are the conclusions drawn adequately supported by the results?

Yes

Are sufficient details of methods and analysis provided to allow replication by others?

Yes

Reviewer Expertise:

Pediatric infectious diseases, Nosocomial infection in pediatric patients. Antimicrobial stewardship.

I confirm that I have read this submission and believe that I have an appropriate level of expertise to confirm that it is of an acceptable scientific standard.

# Peer review: Reviewer response for version 1

**DOI:** 10.21956/wellcomeopenres.18583.r47294

**Published:** 2021-12-22

**Authors:** Ahmed El-Nawawy

**Affiliations:** 1 Department of Pediatrics, Faculty of Medicine, Alexandria University, Alexandria, Egypt

I read with great interest and enthusiasm the manuscript published by John Clark and his colleagues. I also read carefully the valuable comment of Dr Simon and I do agree with her scientific queries. I do believe that this type of informative research should be presented to the Pediatric community in general and to the PICU community more specifically. This research pointed out to the importance of using newer molecular diagnostic multiplex PCR in microbiological diagnosis to reduce the turnover time, which I think that most of us are using by now this technique in PICU.

 Here is my additional observations and queries to be added to those of Dr Simon:

1. I hoped that the setting in which the study is performed to be described shortly.
2. A “RECORD” algorithm could be more representative, although all the necessary data in this context are mentioned. This simply could be replaced.
3. I agree with your statement in the introduction that one hour delay in antimicrobial therapy would reduce survival by 7.6%; so I am just asking why you did not use rapid molecular multiplex PCR in bacterial diagnosis and used it for viral diagnosis?
4. I agree with you that NB-BAL is better than ETT aspirate in microbiological diagnosis, I think in most of PICUs it is a routine by now. Is it possible to add this to your recommendations?
5. The number of viral detections and bacterial detections explained that there must be patients having “mixed infections” which is very common in PICUs. I hope that it will be able to have a table differentiating the three groups: viral, bacterial and mixed, and which is which (which is simply colonization and how to speculate the causative organism).
6. 72/100 patients received antibiotics prior to admission to PICU, and only 50 patients had microbiology and virology tests prior to PICU admission: does this mean that the 22 patients received empirical antibiotics according to the hospital ASP and did not perform any test until PICU admission?
7. Why did molecular viral PCR being reported after 21-25 hours while most of the PCR techniques report in one hour or less according to available machine?
8. It is better to report infections per device day.
9. I repeat the observation of Dr Simon concerning table 6 where B-Lactam is in a separate row and the members of the same group are in separate rows i.e carbapenem and cephalosporin. Usually groups are compared with classes and classes are compared with classes.
10.  The authors reported 21 resistant samples of them 8 patients had MDR resistant organisms, but from table 7 all MDR were 2 organisms at different sites (no XDR & PDR). What is important I to know the resistant genes which carry a significant importance on epidemiological basis. This again emphasizes the importance of multiplex PCR which detects not only the organism but their resistant genes. I have to ask how you managed these resistant organisms.
11. In page 8 the author discussed the difficulty in diagnosing meningoencephalitis. I can’t see why a CSF PCR for bacteria and viruses detection in 45-60 minutes as well as a confirmative MRI will not do.
12. Mortality: I expected to find a statistical analysis for the “Risk factors” of mortality if possible. Moreover it was reported only the PICU mortality and not the 30 days mortality.

To conclude this is an outstanding work, we all seek for perfections. I hope that all the reviewers’ observations could be considered in re-publication which this manuscript deserves.

Is the work clearly and accurately presented and does it cite the current literature?

Yes

If applicable, is the statistical analysis and its interpretation appropriate?

Yes

Are all the source data underlying the results available to ensure full reproducibility?

No source data required

Is the study design appropriate and is the work technically sound?

Yes

Are the conclusions drawn adequately supported by the results?

Yes

Are sufficient details of methods and analysis provided to allow replication by others?

Yes

Reviewer Expertise:

Reviewer expertise: Pediatric Intensive Care – Sepsis & septic shock – Pediatric Infections & in factious disease in PICU – Fluid & Electrolyte management in PICU – Pediatric Mechanical Ventilation.

I confirm that I have read this submission and believe that I have an appropriate level of expertise to confirm that it is of an acceptable scientific standard, however I have significant reservations, as outlined above.

# Response

**Published:** 2022-02-28

**Authors:** John Clark

**Affiliations:** University of Cambridge Addenbrooke's Hospital Cambridge, UK

The authors thank Dr El-Nawawy for the review and address it as follows:

**This research pointed out to the importance of using newer molecular diagnostic multiplex PCR in microbiological diagnosis to reduce the turnover time, which I think that most of us are using by now this technique in PICU.**

 We disagree that the use of multiplex PCR to detect bacterial as well as viral infection is widespread in well-resourced PICU settings, let alone globally. We are aware of routine use of commercially available respiratory multiplex PCR panels that include several viruses, but these have a limited number of atypical bacterial organisms eg. Pertussis and Haemophilus incorporated. On reviewing the literature, we have not identified any centres that are routinely using panels that include a wider range of bacterial targets, hence this requires further evaluation.

**A “RECORD” algorithm could be more representative, although all the necessary data in this context are mentioned. This simply could be replaced.**

 Thank you for the suggestion, we have reviewed the manuscript against the STROBE checklist for observational studies and attempted to address all recommended points.

**I agree with your statement in the introduction that one hour delay in antimicrobial therapy would reduce survival by 7.6%; so I am just asking why you did not use rapid molecular multiplex PCR in bacterial diagnosis and used it for viral diagnosis?**

 Multiplex PCR testing for a variety of commonly detected bacterial organisms is not routinely available in this institution. In the setting of respiratory infection however, it is subject to an ongoing evaluation in this centre. ^1^

 I agree with you that NB-BAL is better than ETT aspirate in microbiological diagnosis, I think in most of PICUs it is a routine by now. Is it possible to add this to your recommendations?

 We disagree – most centres do not undertake NB-BAL routinely, and the procedure was introduced to improve the reliability of the assay during this study. However it must also be noted that the study was not sufficiently powered to evaluate the performance of sampling via NB-BAL versus ETT aspirate, given this collection method was introduced following this project.

**The number of viral detections and bacterial detections explained that there must be patients having “mixed infections” which is very common in PICUs. I hope that it will be able to have a table differentiating the three groups: viral, bacterial and mixed, and which is which (which is simply colonization and how to speculate the causative organism).**

 This study represents a range of indications for admission and subsequent treatment. On CSF samples, no child had a positive test result on microbiology or virology. In addition there were no blood-borne viruses detected. Therefore, we focus here on groupings of patients according to respiratory diagnostic tests. 58/100 patients had both microbiology (any of ETT aspirate/pleural aspirate/NB-BAL culture) and virology testing (any of NP swab, NPA or ETT viral respiratory panel) performed. Of these patients, 36 (62%) had viral detection only, 6 (10%) had bacterial detection only, 11 (19%) had mixed cause and the remaining 5 (9%) had no respiratory microorganism identified. Due to the range of patients in the cohort it is challenging to stratify likelihood of the true cause of illness. For example, for children that were more severely unwell and perhaps immunosuppressed there is a lower threshold for intervention and significance attached to investigations compared to a previously well child.

**72/100 patients received antibiotics prior to admission to PICU, and only 50 patients had microbiology and virology tests prior to PICU admission: does this mean that the 22 patients received empirical antibiotics according to the hospital ASP and did not perform any test until PICU admission?**

 Dr El-Nawawy is correct in this. Some children have not had cultures performed prior to PICU admission despite receiving antibiotics due to administration for surgical prophylaxis or the fact that transfers to PICU may have been time critical eg. Traumatic brain injury, where there was not sufficient time for referring teams to obtain cultures prior to transfer.

**Why did molecular viral PCR being reported after 21-25 hours while most of the PCR techniques report in one hour or less according to available machine?**

 Point of care molecular viral PCR testing is unavailable in this PICU and samples are sent to the diagnostic laboratory. Turnaround time is limited by staffing of this service. Of note, in the setting of the SARS-CoV-2 pandemic, all lower respiratory samples (ETT aspirate and NB-BAL) were initially handled in a containment level 3 laboratory before being split for further microbiological and virology investigations.

**It is better to report infections per device day.**

 Rates of BSI and VAP have been added to the manuscript (Results -> Patients with suspected and confirmed infection).

**I repeat the observation of Dr Simon concerning table 6 where B-Lactam is in a separate row and the members of the same group are in separate rows i.e carbapenem and cephalosporin. Usually groups are compared with classes and classes are compared with classes.**

 Thank you. Table 6 has been updated to classify antimicrobials more clearly.

**The authors reported 21 resistant samples of them 8 patients had MDR resistant organisms, but from table 7 all MDR were 2 organisms at different sites (no XDR & PDR). What is important I to know the resistant genes which carry a significant importance on epidemiological basis. This again emphasizes the importance of multiplex PCR which detects not only the organism but their resistant genes. I have to ask how you managed these resistant organisms.**

 As per reviewer 3 request the table has been removed. We provide some information regarding management of resistant isolates to our reply to reviewer 1. AMR gene testing is not routinely available in this institution however for unusual isolates, samples can be sent to a national reference laboratory. Only one sample in this cohort was sent to the national reference laboratory however it was unable to be processed.

**In page 8 the author discussed the difficulty in diagnosing meningoencephalitis. I can’t see why a CSF PCR for bacteria and viruses detection in 45-60 minutes as well as a confirmative MRI will not do.**

 In this centre meningitis and encephalitis are diagnosed clinically, in addition to testing of blood and CSF. Cell counts on CSF are available within hours of sampling to assist initial rationalisation of treatment however CSF viral PCR testing in this cohort took a median of 39.3 hours. Bacterial PCR of CSF is not routinely available. Neuroimaging would only be performed if there were atypical features related to the presentation, or to investigate for raised intracranial pressure prior to lumbar puncture. This would usually be via CT. MRI is not undertaken routinely due to resources both in the radiology department and having a PICU team available to transport the ventilated patient. In addition it would be unlikely to change management unless undertaken to identify an alternative cause of the patient’s presentation.

**Mortality: I expected to find a statistical analysis for the “Risk factors” of mortality if possible. Moreover it was reported only the PICU mortality and not the 30 days mortality.**

 The focus of the present study was to identify performance of diagnostic tests and describe antimicrobial use in the PICU. The study was not sufficiently powered to undertake an analysis of risk factors for mortality due to the patient group being heterogenous whilst the mortality rate in PICU was low at 6%. It was not possible to determine the 30-day mortality due as this would have required the researchers to contact the patients’ families – which was not permissible when the study was registered as an audit (approved internally). 

^1. ^Clark, J. A. *et al.* Rapid Assay for Sick Children with Acute Lung infection Study (RASCALS): diagnostic cohort study protocol. *BMJ Open* **11**, e056197 (2021).

# Peer review: Reviewer response for version 1

**DOI:** 10.21956/wellcomeopenres.18583.r47378

**Published:** 2021-12-16

**Authors:** Sanjay Patel

**Affiliations:** 1 The British Society for Antimicrobial Chemotherapy, Birmingham, UK

The authors aim to address the extremely important topic of antimicrobial stewardship in severely unwell children admitted to PICU. The study was conducted between 30/10/19 and 19/2/20, during which 100 ventilated children were recruited. Children were identified by the lead clinician and data were collected on their underlying diagnosis, site of presumed infection, choice of antimicrobials and subsequent microbiology/virology results. The majority of children recruited were empirically started on IVAbs; however, only a small proportion was subsequently found to have a positive bacterial or fungal culture result. Based on this funding and the fact that it tool 55 hours/120 hours to get a final negative endotracheal culture/blood culture result, the authors conclude that novel diagnostics are required in the PICU setting.

 Although I agree with this final statement, I am far from convinced that the data presented in this study allows this conclusion to be reached. 

 The main issue I have with this study is that the authors appear to disregard the positive virology samples in their patients and appear to suggest that a negative bacterial culture is required to stop antibiotics. 52 of their admissions were thought to have a LRTI, of which at least 31 had an admission diagnosis of bronchiolitis. Of the 99 respiratory samples sent (ETT or NPA), 72 were positive for a viral pathogen and results were available between 21-24 hours. The principles of antimicrobial stewardship are summarised in START SMART then FOCUS. It is entirely reasonable to start antibiotics in an unwell child presenting with infection; however, it is crucial that at 48 hours, the child is reviewed with their microbiological and virology results, along with their inflammatory markers. The discussion presented within this paper makes it appear that there is little antimicrobial stewardship support available - it would be useful if the authors could clarify whether there is an antimicrobial stewardship service in their hospital. I am surprised by the discussion about needing to wait 120 hours for a final negative blood culture - although this is the time it may take to produce a final negative culture result, it is common practice amongst clinicians to review a blood culture at 48 hours and I am sure that the laboratory will issue a "negative at 48 hours" report. For this reason, I find this a rather disingenuous narrative to support the authors' conclusion about the need for rapid tests.

 Results:  Patients with suspected and confirmed infection p4: "The most common indications for commencing antimicrobials within 48 hours of admission were suspected LRTI (52%), central nervous system (CNS) (29%) and bloodstream infection (19%)" Are these supposed to be percentages or absolute numbers. Table 2 suggests that these are absolute numbers with a denominator of 105 - all the percentages should then be slightly lower than stated.

 Results: Although the authors provide results on the proportion of children admitted with LRTIs having subsequent positive bacteriology results, they do not subdivide this group into those with bronchiolitis (who are unlikely to have a secondary bacterial infection) and those with pneumonia. Of the 9 admissions with pneumonia, how many had a positive tracheal aspirate culture (bacteriology)? How does this compare to the 57% positivity rate in children with VAPS?

 Results: Performance of microbiology and virology investigations undertaken during PICU admission - p4 - paragraph 2 starts with a discussion about viral PCR results but then lists a set of bacterial pathogens. The authors provide no information about the site of these pathogens - are they all from tracheal aspirate samples or do they also include blood culture results?

 AMR results:  I think that table 7 is extremely unhelpful and adds very little to this manuscript. The study was not powered to identify an association between Ab use and AMR rates. If the authors are able to justify the inclusion of AMR data, I suggest that they report rates more conventionally ie MRSA, VRE, ESBLs and CREs (as opposed to trimethoprim resistant E Coli).

 Discussion: In addition to the comments above, I wonder whether the authors should state that one of the reasons that only 17% of children being admitted with a LRTI is that most of them don't have a bacterial infection (and have a confirmed virus instead). In the presence of a robust AMS service, I am not sure that having a rapid diagnostic panel should impact on duration of Ab prescribing in the majority of these children (and will not make any difference to the proportion started empirically on Abs).

Is the work clearly and accurately presented and does it cite the current literature?

Partly

If applicable, is the statistical analysis and its interpretation appropriate?

Not applicable

Are all the source data underlying the results available to ensure full reproducibility?

Yes

Is the study design appropriate and is the work technically sound?

Yes

Are the conclusions drawn adequately supported by the results?

No

Are sufficient details of methods and analysis provided to allow replication by others?

Yes

Reviewer Expertise:

Antimicrobial stewardship and AMR

I confirm that I have read this submission and believe that I have an appropriate level of expertise to state that I do not consider it to be of an acceptable scientific standard, for reasons outlined above.

# Response

**Published:** 2022-02-28

**Authors:** John Clark

**Affiliations:** University of Cambridge Addenbrooke's Hospital Cambridge, UK

The authors thank Dr Patel for his comments. These are addressed in turn:

**The majority of children recruited were empirically started on IVAbs; however, only a small proportion was subsequently found to have a positive bacterial or fungal culture result.** **Based on this funding and the fact that it tool 55 hours/120 hours to get a final negative endotracheal culture/blood culture result, the authors conclude that novel diagnostics are required in the PICU setting. Although I agree with this final statement, I am far from convinced that the data presented in this study allows this conclusion to be reached.**

**The main issue I have with this study is that the authors appear to disregard the positive virology samples in their patients and appear to suggest that a negative bacterial culture is required to stop antibiotics. 52 of their admissions were thought to have a LRTI, of which at least 31 had an admission diagnosis of bronchiolitis. Of the 99 respiratory samples sent (ETT or NPA), 72 were positive for a viral pathogen and results were available between 21-24 hours. The principles of antimicrobial stewardship are summarised in START SMART then FOCUS. It is entirely reasonable to start antibiotics in an unwell child presenting with infection; however, it is crucial that at 48 hours, the child is reviewed with their microbiological and virology results, along with their inflammatory markers. The discussion presented within this paper makes it appear that there is little antimicrobial stewardship support available - it would be useful if the authors could clarify whether there is an antimicrobial stewardship service in their hospital. I am surprised by the discussion about needing to wait 120 hours for a final negative blood culture - although this is the time it may take to produce a final negative culture result, it is common practice amongst clinicians to review a blood culture at 48 hours and I am sure that the laboratory will issue a "negative at 48 hours" report. For this reason, I find this a rather disingenuous narrative to support the authors' conclusion about the need for rapid tests.**

 In this institution, virology results are taken within the context of the patient, the severity of illness and other biochemical and microbiological tests. The present study only reports on children requiring mechanical ventilation in the PICU. Whilst the identification of a virus on a respiratory panel provides some reassurance as to the cause of illness, it is not definitive. This can be due to prolonged shedding of RNA as highlighted by reviewer 1, with the virus no longer active; the detection of a virus that is not actively contributing to a child’s severe presentation (for example some presentations with rhinovirus or adenovirus), or there is a bacterial co-infection. Bacterial co-infection is common in children requiring mechanical ventilation for bronchiolitis. ^2,3^ Preliminary reports are not typically available for microbiology culture of respiratory secretions hence information provided through diagnostic tests is limited in the setting of suspected respiratory infection. Clinical parameters are largely depended on to make antimicrobial decisions.

 In terms of blood culture results, preliminary reports are available and there is a BacT/Alert system in the laboratory. Discussion -> paragraph 2 has been reworded to avoid confusion relating to timing of preliminary review of antimicrobials.

 Support is available in this PICU for antimicrobial decision making. The manuscript has been updated to describe this (Methods -> Paragraph 4).

**Results: Patients with suspected and confirmed infection p4: "The most common indications for commencing antimicrobials within 48 hours of admission were suspected LRTI (52%), central nervous system (CNS) (29%) and bloodstream infection (19%)" Are these supposed to be percentages or absolute numbers. Table 2 suggests that these are absolute numbers with a denominator of 105 - all the percentages should then be slightly lower than stated.**

 These percentages reflect the proportion of patients treated for each of these problems within the first 48 hours of admission. There were some patients who did not receive a culture for the relevant problem described hence the discrepancy with table 2 which reports culture results versus treatment episodes.

**Results: Although the authors provide results on the proportion of children admitted with LRTIs having subsequent positive bacteriology results, they do not subdivide this group into those with bronchiolitis (who are unlikely to have a secondary bacterial infection) and those with pneumonia. Of the 9 admissions with pneumonia, how many had a positive tracheal aspirate culture (bacteriology)? How does this compare to the 57% positivity rate in children with VAPS?**

 Culture was much higher yield in children with VAP (5/7 (72%) culture positive) than those that received a culture at the time of admission for a primary lung infection. Of children that received a ETT culture within 48 hours of admission, 1/27 (3.7%) of bronchiolitis patients and 1/8 (12.5%) of those with bronchiolitis had a positive culture. A likely contributing factor is that 72% of all patients had antimicrobial administration prior to PICU admission. As per the aforementioned studies the expected bacterial co-infection rate in bronchiolitis may be as high as 37%.

**Results: Performance of microbiology and virology investigations undertaken during PICU admission - p4 - paragraph 2 starts with a discussion about viral PCR results but then lists a set of bacterial pathogens. The authors provide no information about the site of these pathogens - are they all from tracheal aspirate samples or do they also include blood culture results?**

 ‘On ETT aspirate culture’ added for clarity (Results -> Performance of microbiology and virology investigations undertaken during PICU admission -> Paragraph 2)

**AMR results: I think that table 7 is extremely unhelpful and adds very little to this manuscript. The study was not powered to identify an association between Ab use and AMR rates. If the authors are able to justify the inclusion of AMR data, I suggest that they report rates more conventionally i.e., MRSA, VRE, ESBLs and CREs (as opposed to trimethoprim resistant E Coli).**

 The table has been removed as per reviewer recommendation.

**Discussion: In addition to the comments above, I wonder whether the authors should state that one of the reasons that only 17% of children being admitted with a LRTI is that most of them don't have a bacterial infection (and have a confirmed virus instead). In the presence of a robust AMS service, I am not sure that having a rapid diagnostic panel should impact on duration of Ab prescribing in the majority of these children (and will not make any difference to the proportion started empirically on Abs).**

 Addition of the following to the manuscript ‘Cultures may also be negative due to no bacteria being present, hence the value of fast turnaround highly sensitive tests for early cessation of antimicrobial therapy’ (Discussion -> Paragraph 3).

 The authors agree that the impact of a highly sensitive multi-pathogen diagnostic panel is unknown in the PICU clinical setting as it is yet to be evaluated. This is the rationale behind the Rapid Assay for Sick Children for Acute Lung infection Study (RASCALS) at this institution (see BMJ Open protocol). ^1^ Given that most children admitted to PICU receive antimicrobial therapy empirically, this assay could be a first step to definitively inform rationalisation or cessation of therapy by ruling out bacterial infection. Whilst most lower respiratory tract infections may well be viral, in clinical practice, children who exhibit severe respiratory failure and/or signs of systemic inflammation or multi-organ disease continue to be treated with antimicrobials until bacterial infection is ruled out definitively. We hope assays such as the one we are evaluating will allow greater confidence for clinicians to de-escalate the use of antimicrobials.

 1.        Clark, J. A. *et al.* Rapid Assay for Sick Children with Acute Lung infection Study (RASCALS): diagnostic cohort study protocol. *BMJ Open* **11**, e056197 (2021).

 2.        Wiegers, H. M. G. *et al.* Bacterial co-infection of the respiratory tract in ventilated children with bronchiolitis; a retrospective cohort study. *BMC Infect. Dis.* **19**, 938 (2019).

 3.        Thorburn, K., Harigopal, S., Reddy, V., Taylor, N. & van Saene, H. K. F. High incidence of pulmonary bacterial co-infection in children with severe respiratory syncytial virus (RSV) bronchiolitis. *Thorax* **61**, 611 LP – 615 (2006).

# Peer review: Reviewer response for version 1

**DOI:** 10.21956/wellcomeopenres.18583.r44127

**Published:** 2021-06-14

**Authors:** Arne Simon

**Affiliations:** 1 Pediatric Hematology and Oncology, Children's Hospital Medical Center, University Hospital of Saarland, Homburg, Germany

This single center prospective observational study designed and accomplished by John Clark and coworkers is methodically adequate, well presented and it adds important information to our understanding concerning the obstacles to perform targeted antimicrobial treatment in pediatric intensive care patients on mechanical ventilation.

 At the institution of the authors, an antibiotic stewardship team is in charge and there are diagnostic and therapeutic internal guidelines addressing the management of nosocomial infections in pediatric intensive care patients.

 To my knowledge, the problem of low yield and long turnaround time of microbiological (including virological) diagnostics in this vulnerable population has not been described in such detail.

 72 of 100 patients included in this study have received antibiotics before admission to the PICU. This may in part explain the low yield of bacterial cultures. The highest yield was found in respiratory (rt) PCR investigations for viral pathogens. Perhaps the authors should comment on:

- The quite long turnaround time of 22 hours for these PCRs
- the idea to perform point of care testing in this high acuity patients concerning the most important viral pathogens (such as influenza and RSV - beneath SARS-CoV-2 now)
- the fact, that not every positive PCR is confirmative for a causative role of this particular virus (e.g. due to the fact, that PCR results may be positive for weeks after primary infection in this age group)

Concerning the description of the epidemiology of the nosocomial infections, it would be informative to describe the rates per 1000 utilization days (BSI → central line days; VAP → days on mechanical ventilation) to make these results more comparable to other studies.

 I do not totally agree with the statement on page 6, that “It is common practice to await microbiology results prior to cessation of antimicrobial therapy…” since the suspicion of a VAP may be questioned after 24 to 48 hours in patients with clinical and radiological signs which can be allocated to other reasons.

 Tab. 6 differentiates between “Beta lactam”, carbapenem and cephalosporin although carbapenems and cephalosporins are betalactam antibiotics. It would be interesting to know, which antibiotics are summarized to “Beta Lactam” (Ampicillin-Sulbactam? Piperacillin with or without Tazobactam?)

 Referring to the median age of the population and the nosocomial setting, it is unclear, why these children have received macrolides on 18.7% of all inpatient days.

 The low share of glycopeptides in Tab 6 contrasts with the detection of MRSA and MRSE in 8 patients (Tab 7). Have these patients received daptomycin or linezolid?

 In addition, this list of resistant isolates contains 4 patients with Carbapenem-resistant *Gram*-negative Isolates. How were these patients treated?

 Taken together, these are only minor points and I‘d really appreciate if this study is republished soon after minor Revision.

Is the work clearly and accurately presented and does it cite the current literature?

Yes

If applicable, is the statistical analysis and its interpretation appropriate?

Yes

Are all the source data underlying the results available to ensure full reproducibility?

No source data required

Is the study design appropriate and is the work technically sound?

Yes

Are the conclusions drawn adequately supported by the results?

Yes

Are sufficient details of methods and analysis provided to allow replication by others?

Yes

Reviewer Expertise:

Pediatric infectious diseases, Nosocomial infection in pediatric patients. Antimicrobial stewardship.

I confirm that I have read this submission and believe that I have an appropriate level of expertise to confirm that it is of an acceptable scientific standard, however I have significant reservations, as outlined above.

# Response

**Published:** 2022-02-28

**Authors:** John Clark

**Affiliations:** University of Cambridge Addenbrooke's Hospital Cambridge, UK

We thank Dr Simon for the review comments. Our responses to each point are listed below.

**The highest yield was found in respiratory (rt) PCR investigations for viral pathogens. Perhaps the authors should comment on: **

- **The quite long turnaround time of 22 hours for these PCRs **
- **The idea to perform point of care testing in this high acuity patients concerning the most important viral pathogens (such as influenza and RSV - beneath SARS-CoV-2 now)**
- **The fact, that not every positive PCR is confirmative for a causative role of this particular virus (e.g. due to the fact, that PCR results may be positive for weeks after primary infection in this age group) **

During and prior to the study, point of care PCR testing for respiratory pathogens was not available within the paediatric intensive care unit. This contributed to longer turnaround times than might be expected. Subsequent to the project, BioFire Respiratory 2.1 Panel testing (Biomérieux, France) has been introduced. This panel includes a target for SARS-CoV-2. In addition, the trust introduced Simple AMplification-Based Assay (SAMBA) II SARS-CoV-2 testing.

 The authors agree that positive viral PCR detection does not necessarily confirm an underlying diagnosis due to prolonged RNA shedding after some pathogens are no longer causing symptoms or were an incidental finding.

**Concerning the description of the epidemiology of the nosocomial infections, it would be informative to describe the rates per 1000 utilization days (BSI → central line days; VAP → days on mechanical ventilation) to make these results more comparable to other studies.**

 Rates of BSI and VAP have been added to the manuscript (Results -> Patients with suspected and confirmed infection). These rates need to be interpreted with caution due to variation in the definition used, particularly microbiological definitions, for these problems internationally. In this paper, we have included any instance of commencement or change of antimicrobial to treat for BSI or VAP.

**I do not totally agree with the statement on page 6, that “It is common practice to await microbiology results prior to cessation of antimicrobial therapy...” since the suspicion of a VAP may be questioned after 24 to 48 hours in patients with clinical and radiological signs which can be allocated to other reasons.**

 This section has been reworded for clarity.

**Tab. 6 differentiates between “Beta lactam”, carbapenem and cephalosporin although carbapenems and cephalosporins are beta-lactam antibiotics. It would be interesting to know, which antibiotics are summarized to “Beta Lactam” (Ampicillin-Sulbactam? Piperacillin with or without Tazobactam?)**

 Table 6 has been updated to classify antimicrobials more clearly. The most commonly used antimicrobial of the beta-lactams was ceftriaxone followed by piperacillin-tazobactam. Some subsequent courses of the same antimicrobial were noted by the lead author on repeating the analysis that were not included in the primary manuscript. This has been corrected in the present manuscript –apologies for this error that under reported antimicrobial use.

**Referring to the median age of the population and the nosocomial setting, it is unclear, why these children have received macrolides on 18.7% of all inpatient days.**

 There were two main indications for macrolide treatment. Clarithromycin was given for 114 PICU days as a second agent to cover for atypical severe respiratory infection. There were 43 PICU treatment days with azithromycin, and this agent is sometimes used in this region as prophylaxis in children with chronic lung disease with recurrent respiratory infection.

**The low share of glycopeptides in Tab 6 contrasts with the detection of MRSA and MRSE in 8 patients (Tab 7). Have these patients received daptomycin or linezolid?** **In addition, this list of resistant isolates contains 4 patients with Carbapenem-resistant *Gram*- negative Isolates. How were these patients treated?**

 MRSA was detected on routine screening tests in (Study ID 033, 099 and 101). In this centre, patients receive standard decolonisation treatment with mupirocin. No patients in the cohort received daptomycin. Linezolid was used for one patient (Study ID 046) for treatment of presumed CNS infection in the setting of status epilepticus and a ventriculoperitoneal shunt. This was due to previous red man syndrome in response to vancomycin.

 Study ID 32 – had an *AmpC* producing *Citrobacter freundii* on ETT aspirate culture. This was sensitive to ciprofloxacin, gentamicin and meropenem and resistant to amoxicillin-clavulanate. After seven days of piperacillin-tazobactam, treatment was switched to meropenem which was given for eight days.

 Study ID 73 – *Pseudomonas aeruginosa* grew on ETT aspirate culture which was resistant to ceftazidime and piperacillin-tazobactam but sensitive to meropenem. Treatment was changed from piperacillin-tazobactam to meropenem accordingly.

 Study ID 79 – An *AmpC* producing *Enterobacter cloacae* was grown on ETT aspirate culture. This isolate was resistant to cefotaxime, ceftazidime, ertapenem, piperacillin-tazobactam and aztreonam. It was sensitive to ciprofloxacin and gentamicin. This patient received four days of ceftriaxone and three days of fluconazole in PICU before being discharged to the ward due to clinical improvement, prior to microbiology results becoming available.

 Study ID 81 – This patient had an *AmpC* producing *Enterobacter cloacae *on ETT aspirate culture. This was resistant to cefotaxime, piperacillin-tazobactam, aztreonam, ceftazidime and ertapenem. It was sensitive to ciprofloxacin, gentamicin and meropenem. This was initially treated with six days of ceftriaxone and clarithromycin and was changed to an eight day course of meropenem after sensitivity results.

 Study ID 88 – This patient had persistent growth of multi-resistant *Pseudomonas aeruginosa *on ETT aspirate culture. Resistance included ciprofloxacin, meropenem and piperacillin-tazobactam but there was sensitivity to ceftolozane. This patient received 10 different antibiotics during their admission, however the core treatment received was 33 days of tobramycin, 17 days of ceftolozane, 11 days of ciprofloxacin and eight days of colomycin.

 The data presented only records antimicrobials administered during PICU admission – not the subsequent administration following stepdown to the ward. Treatment after PICU admission was not evaluated due to a large proportion of children being transferred back to their local hospital. The authors did not have access to these medical records. The overall effect of this is that the overall duration of glycopeptide treatment is lower than might be expected.
